# Design and implementation of the mobility assessment tool: software description

**DOI:** 10.1186/1472-6947-13-73

**Published:** 2013-07-23

**Authors:** Ryan T Barnard, Anthony P Marsh, Walter Jack Rejeski, Anthony Pecorella, Edward H Ip

**Affiliations:** 1 Department of Biostatistical Sciences, Public Health Sciences, Wake Forest School of Medicine, Winston-Salem, North Carolina, USA; 2 Department of Health and Exercise Science, Wake Forest University, Winston-Salem, North Carolina, USA

## Abstract

**Background:**

In previous work, we described the development of an 81-item video-animated tool for assessing mobility. In response to criticism levied during a pilot study of this tool, we sought to develop a new version built upon a flexible framework for designing and administering the instrument.

**Results:**

Rather than constructing a self-contained software application with a hard-coded instrument, we designed an XML schema capable of describing a variety of psychometric instruments. The new version of our video-animated assessment tool was then defined fully within the context of a compliant XML document. Two software applications—one built in Java, the other in Objective-C for the Apple iPad—were then built that could present the instrument described in the XML document and collect participants’ responses. Separating the instrument’s definition from the software application implementing it allowed for rapid iteration and easy, reliable definition of variations.

**Conclusions:**

Defining instruments in a software-independent XML document simplifies the process of defining instruments and variations and allows a single instrument to be deployed on as many platforms as there are software applications capable of interpreting the instrument, thereby broadening the potential target audience for the instrument. Continued work will be done to further specify and refine this type of instrument specification with a focus on spurring adoption by researchers in gerontology and geriatric medicine.

## Background

**Figure 1:**
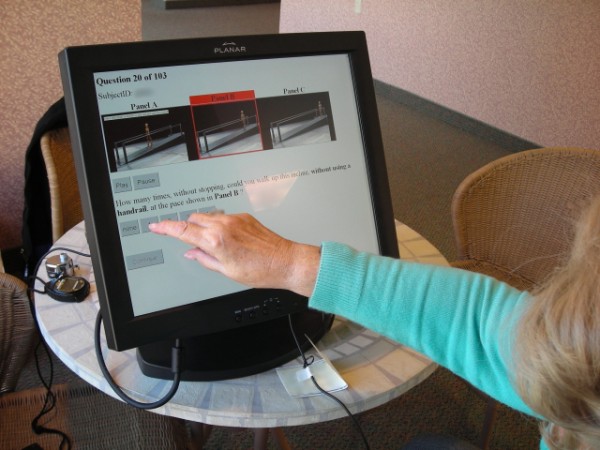
**Touchscreen interface.** A study participant interacting with the touchscreen to answer the questions accompanying each set of animations.

In previous work [1,2], we developed and described an 81-item video-animated tool for assessing self-perception of mobility, which we have called the Mobility Assessment Tool (MAT). Items in the MAT consist of a video and a corresponding measurement item. The videos depict a wooden mannequin performing a wide range of physical activities, and the measurement item consists of a question about the participant’s ability to perform the task, measured on a discrete scale. Participants interact with the software via a capacitive touch screen (Figure 1).

In response to criticisms levied against MAT in a pilot study, we sought to develop a new version of the tool, but because we also anticipated multiple subsequent variations—as well as, potentially, a host of additional instruments using the same (or a similar) structure—we elected to construct a flexible framework upon which new versions of MAT could be implemented.

### Motivation for the MAT family of software

In the original version of the testing software, everything was constructed largely by hand as a series of barely-connected web pages, video files, and scripts. This approach worked perfectly for the pilot study as it was very quick and easy to construct, but it was also fragile and tedious to perform significant changes. Any variation in the number or order of the test items would require (a) the web forms for every affected item (along with their immediately preceding and succeeding neighbors) to be hand-edited and tested, (b) the processing scripts to be updated to reflect the new instrument, and (c) the database schema to be rebuilt.

Though acceptable for the short-lived pilot study, these design deficiencies were not acceptable in the context of the project growing and maturing. In particular, the software behind the new MAT projects needed to permit fast, painless, and reliable manipulation of the instrument, including the ability to easily retain the original test and any other variations without requiring massive duplication of shared resources.

The MAT family of software arose from these simple requirements, providing the following key components: 

1. A common XML-based data file describing all of the important properties of the instrument,

2. An application written in Java to support participants using Mac, Windows, and Linux computers,

3. An application written in Objective-C for participants with access to Apple iPad devices, and

4. A workflow for very quickly and reliably producing customized versions of both the Java and the iOS applications from the source instrument.

### MAT software requirements and architecture

The success of the pilot study and similar work in the past motivated us to build a solution to the above needs that is more flexible than simply meeting the minimum requirements. We envisioned using a test platform with the capabilities described above in several pending future projects, and thus planned the MAT tool accordingly. In particular, while designing the software, we sought to satisfy two philosophical objectives: 

1. Build a system that is sufficiently extensible that it can handle a variety of similar studies without redesigning the software, and

2. Avoid designing a tool so general that there is resultant loss in sensitivity in the assessment process or that requires exorbitant resources to design and maintain.

#### Requirements

The central capability is the ability to define a complete psychometric measurement instrument. Such an instrument must be composed of: 

1. A set of test items, consisting of (a) a question with text and a video or image accompanying it and (b) a discrete set of possible participant responses, where each response is composed of a textual label and a numeric value associated with it;

2. A set of instructions, including possible example questions, that would precede the test items; and

3. An explicitly defined scoring mechanism to automatically associate a numeric value with a particular sequence of responses.

While all studies we intended to support would include requirement 1, requirements 2 and 3 should be optional so that the system is capable of handling them, but does not at any point *depend* upon them to function. The needs of these types of instruments are fairly uniform within the aims we set out to achieve: 

1. Respondents can be required to provide non-scored identifiers before beginning to allow correlation with externally gathered data,

2. The ability to control test presentation policies—such as requiring an item video to be viewed to completion before permitting the participant to respond—that may vary on a study-to-study basis rather than being hard-coded into the software,

3. Stored data for a single run of the test for a single participant should include: (a) any identifiers the participant provided, (b) the value of each individual response, (c) the participant’s overall score, if computed, and (d) the time, date, and duration of the test.

4. All recorded test data should be retrievable as a single table for use and analysis by non-technical researchers instead of just providing, for example, an XML file or a relational database and expecting the researchers, who may have no experience in such technical matters, to retrieve and format the data as they need.

5. Studies are likely to be performed in multiple countries in multiple languages, so internationalization and localization features should be incorporated as fundamental design aspects of the system.

Beyond these 5 critical needs, the ability for the tool to automatically submit responses to a central server would dramatically aid in collecting and organizing response data in large studies. Finally, heterogeneity across varying sites suggests a strong need to build a system with a good deal of interoperability between other systems.

#### Architecture

The resulting MAT software that we describe in this paper successfully meets all of the described objectives and requirements discussed above. In this section we discuss the architecture we have developed that underlies the actual implementations we have produced.

**Figure 2:**
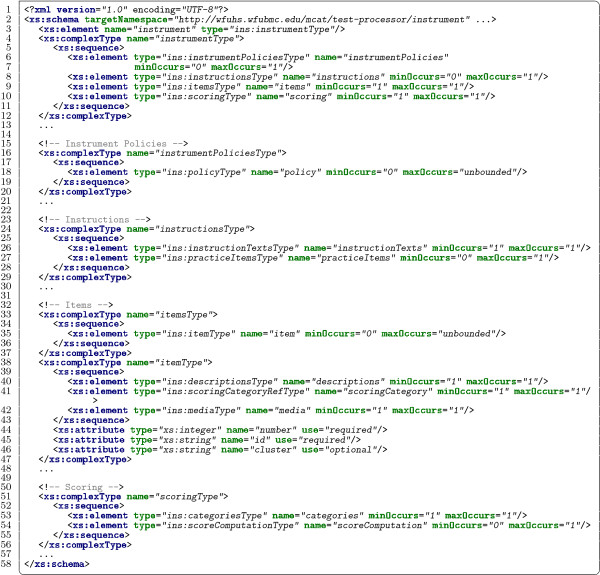
Abridged XML schema for instrument definitions.

**Figure 3:**
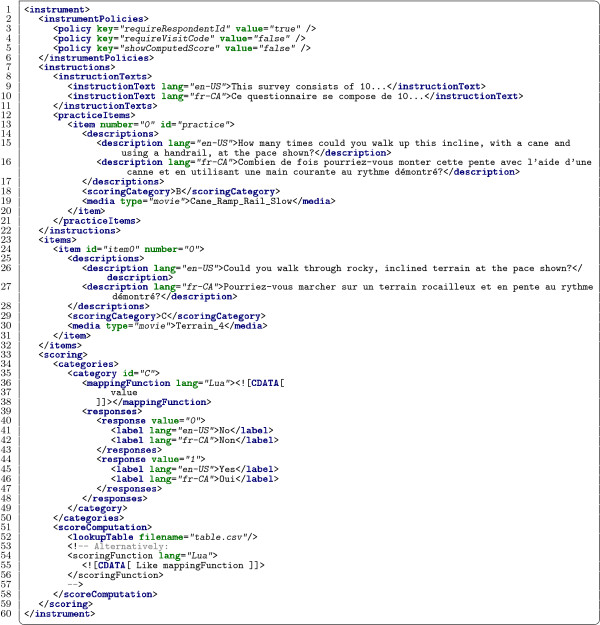
**Minimal instrument XML example.** An example instrument configuration using the MAT XML schema.

The most basic desire that we were able to identify was a need to separate the structure and content of measurement instruments from the software presenting those instruments and collecting responses. This naturally suggests a layer of abstraction between software and data, and guided by this observation we designed an XML schema that would allow us to expressively describe a complete measurement instrument, including localizations and specific study requirements, without having to write any actual code. Confidence in this decision was bolstered by the successful use of XML for various applications throughout healthcare and medical informatics [3-7], especially when structured by an appropriate XML schema [4,8]. Figure 2 provides a partial listing of the schema and Figure 3 provides a minimal example XML document exemplifying that schema.

It is important to note here that this architecture allows an instrument to be modified by altering the content of its associated configuration XML file, and that, although the software implementation that runs the instrument need not be recompiled to reflect those changes, no specification or requirement is provided in support of user modifications at runtime. For our purposes, it is sufficient that a software developer be able to quickly create instrument variations involving only modification of the configuration XML file, but each instrument variation may be distributed as a complete, self-contained application. This approach also simplifies tracking of instrument variations in version control systems, as only a single XML file need change.

#### Instrument schema

Our schema is built upon the notion of the instrument as the root concept, which includes 4 children describing the constituent components of an instrument: instrumentPolicies, instructions, items, and scoring. Throughout the schema there are many elements that contain human-readable text that is intended to be displayed to study participants. Each such element has an optional attribute called *lang* that specifies which localization that text applies to. If the *lang* attribute is absent, the element is assumed to be the default localization to display in the event the system locale is not available. (It is an error for more than one element to be the default.) The elements to which this attribute applies are listed in Table 1.

**Table 1:** Elements of the instrument specification which support multiple languages

	
instructionText	The text that should be displayed when presenting usage instructions to the participant.
description	The textual description that accompanies an instrument item; usually in the form of a question.
label	The textual label associated with one of the possible item responses.

##### instrumentPolicies

The instrumentPolicies section is a simple key-value list of properties defining certain policies of the instrument’s presentation (see Table 2 for a list of configurable policies).

**Table 2:** **Instrument policies that can be configured in the ****instrumentPolicies****section of the instrument specification**

**Policy key**	**Policy type**	**Description**
requireRespondentId	boolean	Indicates whether participants are required to enter a personal identification code before beginning the test.
requireVisitCode	boolean	Indicates whether participants are required to enter a visit code before beginning the test.
requireVideoViewing	boolean	Indicates whether participants must view the entire animation before being permitted to respond.
startVideoAutomatically	boolean	Indicates whether the video should automatically begin playing.
serverSubmissionURL	URL	Specifies the URL of the server to which collected data should automatically be submitted.
showComputedScore	boolean	Indicates whether the computed score (if applicable) should be displayed to the participant following completion of the instrument.

Each boolean-valued policy defaults to **false** and serverSubmissionURL defaults to an empty string.

##### Instructions

The Instructions section provides a mechanism for defining the instructions and accompanying practice test items that will be presented to a participant prior to the actual test. instructionText is just a textual element containing the actual instructions to present. The practiceItems child is identical in structure to the items child of instrument described below.

##### Items

The Items section contains a sequence of item elements, each of which defines a single test item. Test items contain the required attributes *id* and *number*. The former is an arbitrary, unique string for identifying that item uniquely regardless of its position in the test, and *number* is a 0-based index indicating the order in which it appears in the instrument. The description child element contains a list of textual item descriptions which will usually be the text of the questions themselves. media identifies any media item that is to be presented alongside the item text, such as a video clip or an image. Note here that the value of media is *not* a filename but rather an identifying key from which the test application can determine the appropriate media file based on the platform and language.

Finally, scoringCategory is a reference to one of the children of scoring. Because most studies will consist of many items that all share a common set of legal responses, we have elected to define those responses separately from the items they belong to, instead permitting the items to simply indicate which category of responses is appropriate for it. Though this minimizes repetition and ensures consistency, this decision could be considered a drawback in the worst-case scenario that each item is permitted a distinct set of responses. In this case, the logical separation between the item definition and its corresponding response definition could lead to confusion, but it is our belief that such cases are very rare if existing at all among the audience of possible users of our system.

##### Scoring

The Scoring section contains a required categories element and an optional scoreCom- putation element. The children of categories each describe one of the possible categories of item responses referenced by test items. In addition to the id element used to uniquely identify the categories, each category contains a set of responses and an optional mappingFunction. The set of responses lists each possible response that the participant can select along with the *id* that response represents. Note that *id*s must be unique; association between a response *id* and the numeric value that it represents is defined by the mappingFunction. The label portion may be defined in one of two ways: as an attribute of the response element itself (in the case that the label is common across all supported localizations) or as a list of children, where each child specifies the relevant language.

The mappingFunction is different from the other elements discussed thus far. Because responses are often “binned” into a smaller number of categories, the mappingFunction provides a mechanism for arbitrarily associating the unique response id to a numeric value. Currently, the mapping function supports simple expressions manipulating a variable named value, which represents the numeric value of a response. Should the need for additional functionality arise, interpretation of the mapping function could be performed using a domain-specific language built on a more sophisticated, embeddable scripting environment such as Lua.

Instruments that support automatic scoring by the test application can define their scoring mechanism under scoreComputation, using either the lookupTable or scoringFunction element. The scoring method in use in the MAT uses proprietary software (MULTILOG from Scientific Software International, Inc.) to compute IRT item parameters and thus the scores for any sequence of responses. Because we are prohibited by licensing restrictions from distributing the MULTILOG software, we instead computed a lookup table of all possible response sequences. This lookup table is referenced in the lookupTable element, which provides the filename of a two-column table containing a list of all possible combinations of response values for the instrument in the first column and the real-valued score it maps to.

Construction of an exhaustive lookup table is only appropriate for instruments having a sufficiently small space of possible responses that their enumeration and pre-computation is not prohibitively time- or space-consuming. Other instruments can use the scoringFunction to calculate the score at runtime using the same built-in scripting support as mappingFunction, thus permitting the score to be calculated by completing a computation, invoking a web service, or performing some other function in the domain-specific language. Note here that need has not yet arisen for such sophisticated functionality, and thus, at present, the scoringFunction and mappingFunction features support only trivial arithmetic expressions as a proof-of-concept.

#### Test application architecture

The described instrument schema only addresses the XML files that would define an instrument, and the task of actually presenting that instrument to participants and collecting their responses for later analysis requires separate construction of a software application.

Our immediate needs for continuation and expansion of the Mobility Assessment Tool dictated support for both desktop computers (running either Windows or Mac OS X) and Apple iPad devices, and the software we built for each platform shares a common 3-layer architecture consisting of a Presentation layer that interacts with the user, a Logic layer that handles the tasks of managing test execution and progress, and a Data layer that handles interpreting the instrument definition XML files and management of collected user data.

Again, note that no runtime, user-friendly manipulation of the instrument is included among these requirements and specifications.

##### Presentation

The presentation layer is the layer that directly interacts with the user. It supports two user activities: the test activity and the score viewing activity. The former provides a mechanism for the user to provide whatever demographic data is required, optionally present the instructions and practice test items, display instrument items and wait for the participant’s feedback, and finally to pass the participant’s responses to the Logic layer.

The score viewing activity lists previous tests that have been completed and permits examination, for each participant, of each response provided and their overall score. It also provides the tools for exporting scores to simple table files that can be examined in external software.

##### Logic

The logic layer performs management functions between the presentation and data layers, ensuring that tests conform to required policies and that only valid data is presented to the user or stored in the database. In particular, the responsibilities of the logic layer include: ensuring that any required demographics are collected from the participant before presenting the test, iterating through test items from beginning to end in the correct order (and possibly discarding the test if incomplete), validating and recording data collected from participants’ test sessions, computing the score for completed tests as required, and (if appropriate) ensuring that tests are eventually submitted to a central server even if submission fails at the time a test is completed.

##### Data

Lastly, the data layer handles the technical tasks related to marshalling and unmarshalling the data structures that the presentation and logic layers depend upon to and from disk, database, or web server. In particular, the responsibilities of the data layer include: interpretation of dataset files in as robust a fashion as possible; providing to the logic layer, when requested, the appropriate localization of human-readable text elements; communicating with the central server and actually performing the data transmissions requested by the logic layer; and automatically keeping backup archives of user’s test responses.

The data layer and its implementation are intimately dependent upon particulars of the instrument schema and as the instrument schema evolves, so too must the support mechanisms in place in the data layer. The current schema (and implemented data layers) have been stable through several versions of the MAT software, and it is expected to remain stable for the foreseeable future. However, the MAT architecture is not intended to be a one-size-fits-all solution to computerized instruments, so significant modifications would be necessary to cope with significant departures from the assumptions of MAT.

## Implementation

As discussed in the previous section, the Mobility Assessment Tool needed to be implemented for both desktop computers (running either Windows or Mac OS X) and Apple iPad devices, and the disparity between these two platforms precluded the use of the same software for both. Due to greater urgency, the Windows/Mac version was first implemented nearly a year earlier than the iPad version, and as such it is a considerably more mature product. In spite of that, the core functionality was easily provided on both platforms, each of which perform identical functions using the same instrument definition XML file, thus demonstrating the viability of our instrument schema for defining an interactive measurement instrument independently of the instrument’s actual implementation.

The test applications themselves are very simple software applications that conform to the requirements previously discussed and require no unusual expertise to implement. As such, details of implementation are omitted here; instead, this section only highlights a few noteworthy design decisions and implementation issues for each application. (Readers wishing to learn more about these applications may visit http://mat-sf.wfuhs.arane.us/.)

### Java implementation

The requirement of providing functionality on both Windows and Mac OS X machines naturally leads to the decision to build an application using a cross-platform language. We elected to use Java due to its maturity, ubiquity, and the vast collection of freely available third-party libraries with compatible licensing terms.

The Java-based MAT implementation depends particularly heavily upon the Spring libraries and several libraries from Apache Commons as a foundation. XStream was instrumental in quickly building a stable and reliable data layer, as it handles all responsibilities related to marshalling and unmarshalling the Java objects representing response data. The ability to export response data tables to Excel was provided by JExcelApi. And finally, Apache Commons HttpClient transmitted data to the collection server.

#### Video playback

Video playback provided the most substantial hurdle in building a stable, reliable application. Initially, we depended upon QTJava, a Java wrapper around Apple’s QuickTime software, to decode and play the animations. Unfortunately, we discovered that QTJava is a deprecated library [9], and that only 32-bit versions of the native binaries that act as the gateway between the Java portion of the library and QuickTime are available. Because of this, users on 64-bit machines would have to ensure that they had a 32-bit Java Runtime Environment (JRE) installed even if a 64-bit JRE was already installed, which led to a considerable amount of time lost providing basic support.

Because of this and other difficulties with QTJava, we elected to switch to a 100% Java-based video playback library to ensure that incompatibilities regarding native libraries would not cause problems. We eventually chose to build upon Cortado, an open-source, cross-platform, Java-based video playback library.

The Cortado library was used largely intact, but a number of important modifications were made to the playback subsystem. In particular, a number of non-essential—for MAT—features such as audio playback and subtitle support were removed in order to reduce CPU load and improve framerate. Additionally, the playback and buffering mechanisms were adjusted so that playback of video files from the local filesystem, from a JAR file, or from a web resource would all behave identically. And finally, support for directly retrieving (and changing) the playback position of a video—again, irrespective of the video’s data source— were added.

Due to the ease of format conversion offered by tools such as ffmpeg and the highly modular nature of our Java implementation, switching from QuickTime MOV to Ogg Theora videos (as required by Cortado) was without incident, and the newest version of our Java-based MAT software appears to run perfectly on Mac OS X, Windows, and Linux.

### Apple iPad implementation

The iPad version of the MAT software was motivated by a desire for a mobile implementation of the tool. The desire to use a widely available, commercial, off-the-shelf (COTS) device with a touchscreen interface, extensive networking capabilities, and an established community of developers led to a choice between the Apple iOS platform and the Google Android platform. Positive institutional experience with iOS coupled with a preference to avoid the implementation issues inherent in any system with significant heterogeneity motivated the decision to target iOS. Though the similarities between the iPad and the iPhone/iPod Touch devices far exceed their differences, the compatibility between the iPad’s larger display and the practical needs of a geriatric participant population dictated our final decision to support only the larger device.

Whereas the Java application relied upon the use of numerous third-party libraries, the iPad application depended almost exclusively upon frameworks native to iOS, with a single external dependency upon MGSplitViewController to enhance the appearance and ease-of-use of a single interface element.

The iPad data layer was implemented using a variety of built-in frameworks. The built-in functionality provided by Core Data simplified persistence of collected responses. Response data was transmitted to the server using the URL loading system of the Foundation framework.

At the time of writing, the iPad MAT software is being successfully implemented in three studies and is fundamentally complete with respect to the requirements of presenting the measurement instrument and collecting responses. However, the only present mechanism for retrieving collected data from the device is to enable submission of results to a central server, thus preventing its use when such a server is unavailable or inappropriate. Additional functionality will be provided in subsequent iterations of the software.

### Implementation comparison

**Figure 4:**
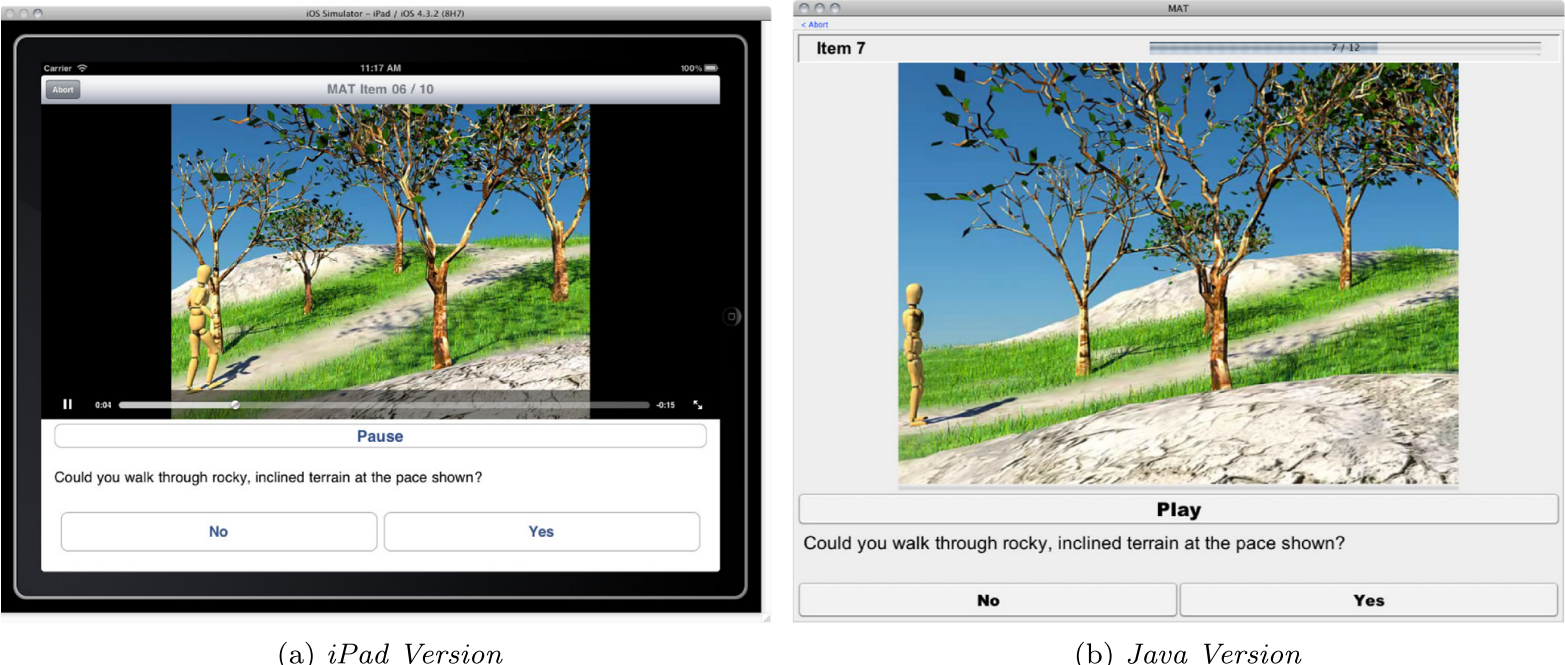
**iPad and Java Implementations, Side-By-Side.** Screenshots of the iPad—left, in panel **(a)**—and Java—right, in panel **(b)**—implementations of the MAT software.

The iPad implementation of the MAT software offers nearly all of the functionality of its more mature counterpart (Figure 4), but required substantially less time to complete. Whereas the first fully-functional Java implementation required roughly 7 working days to complete, the iPad implentation consumed only 1.5 days. The developers attribute this primarily to (a) the simplicity of targeting a locked-down, homogeneous device and (b) the availability of OS-level APIs that provide support for building consistent user interfaces, performing video playback, marshalling and unmarshalling data, and communicating with the server. Though greater familiarity with the problem domain likely accounted for some portion of that speedup, the number of lines of code in each application is a valid, concrete metric that can help quantify the relative simplicity of the iPad implementation [10-12]. After retrieving from the version control system used during development a version of the Java application with an identical feature set, we calculated 8566 lines of Java code in the Java application and 3527 lines of Objective-C code in the iPad application.

It is important to note that this comparison does not offer generalizable conclusions regarding the suitability of iOS-targeted Objective-C over Java or vice-versa. Rather, it is presented here as a noteworthy observation as it may have implications for the cost of continued development and maintenance of the MAT software in the future and as such is an important metric to consider when examining the outcome of our efforts.

## Results and discussion

The process of designing the successor to the original pilot study has been underway for over a year and within that span we have successfully constructed two different implementations of the testing software that satisfy our initial objectives. Additionally, both versions are virtually identical from a participant’s point-of-view and literally identical from the perspective of specifying the instrument, as the same instrument XML file can be used for both versions.

The Java application is currently being used in two separate studies while the iPad application is being implemented in three others. Additionally, because of the simplicity of defining instrument variations, we have been able to accommodate requests for specific alterations with a turnaround time bound by the effort involved in creating new media, effectively eliminating the difficulty of modifying the instrument as a barrier to experimentation and adaptation.

Use of a common instrument specification that is separated from the instrument implementation has the added benefit that an instrument can be immediately used on any platform for which the test application software has been written, with no additional burden to the study designer, thus potentially expanding pool of eligible study sites.

### Computerized adaptive testing

The success of the instrument specification and test application software has already led to plans for its continuation and expansion.

First, we have begun implementation of a computerized adaptive test (CAT) for MAT on the iPad platform. MAT presents a static, pre-defined set of items out of the original 81. These items were selected by careful, manual analysis of the data obtained through the 81-question long-form pilot study. However, an active area of investigation is the use of CAT for instruments in the health domain [13,14]. For example, the Patient Reported Outcomes Measurement Information System (PROMIS) provides a range of CAT instruments that cover several domains of health including physical, mental, and social health [15]. An important advantage of CAT implementation is that it can bring a substantial reduction in patient response burden, as well as alleviating both ceiling and floor effects, which are sometimes seen in traditional mode of assessments.

Though not yet implemented, the MAT architecture provides a flexible framework for allowing the incorporation of one (or more) adaptive approaches.

One challenge in computerized adaptive testing is the scoring of participants and the selection of items. Techniques such as maximum likelihood and maximum a posteriori often incur a significant computational penalty after every item, which is not practical for deployment onto mobile and low-cost computing platforms. Existing CAT tools suffer from these limitations and are consequently unsuitable for IRT-based instruments like MAT. We thus seek to implement a MAT-CAT that overcomes these obstacles while retaining the significant contextual advantages conferred by the MAT video animations.

To address this constraint, we have begun exploration of a fixed-length CAT implementation of MAT and a psychometric evaluation of the tool has been recently reported [16]. We have also begun testing of a pre-computed tree of possible fixed-length, adaptive presentations of the instrument. In this approach, the set of all possible permutations of *n* items drawn from an *m*-item question bank is generated, with the score and standard error computed for each of the *i* ∈ {1,2,…,*n*} items in each possible sequence. This naturally corresponds to a binary tree structure with depth *n* and 2^*n*+1^ − 1 nodes. Each node in the tree represents an item presented to the user, with the root of the tree representing the first presented item, which every respondent answers. The left child of a node corresponds to the next question asked if the participant responds in the negative, and the right child corresponds to the next question asked if the participant responds in the affirmative. Each node—save the root—also references the score and standard error that the respondent has attained based on the preceding responses, and the values associated with the leaf nodes are the final scores for the traversal from the root.

Though pre-computation of the CAT tree requires the tree data to be embedded in the instrument configuration, it offers several distinct advantages in the often resource-constrained MAT deployment contexts. First, inclusion of this CAT algorithm requires only minor changes in the logic layer of the implementations and trivial changes to the instrument schema (to reference the tree data). This facilitates rapid inclusion in any and all MAT implementations.

Second, retrieval of item *i* + 1 after a user has responded to item *i* requires only traversal of the binary tree to depth *i* + 1, which is bound by *O*(log_2_ 2^*i*+1^) = *O*(*i* + 1) = *O*(*i*). (Note that, in practice, the computational complexity will be *O*(1) (constant time) by simply storing the tree position at each item instead of re-traversing from the root.) The pre-computation approach does have at least one significant limitation however: a tree representing the possible instruments of length *n* will contain 2^*n*+1^ − 1 nodes, which grows with *O*(2^*n*+1^). This exponential growth means that pre-computation of instruments containing more than around 20 items represents an effectively intractable barrier, both in terms of the pre-computation cost and in terms of the runtime storage requirements. However, our experience showed that there were only marginal gains in accuracy after 10–12 items, thus allowing the CAT version of MAT to be safely terminated at 12 or fewer items [16].

Because MAT-CAT is an active avenue of investigation, its implementation requirements and constraints are very much in flux. However, it is inevitable that incorporating this (or any) form of CAT into the MAT framework will require augmentation of the instrument schema and the data and logic layers of the implementation. However, because CAT can be envisioned as little more than a change in instrument flow (from static sequential to dynamic adaptive), existing architectural elements need not change, and CAT can be introduced as just a new layer atop the established schema.

### Digitally packaged instrument

The success of decoupling the instrument software from the instrument specification has motivated us to create a more flexible and general model for developing and disseminating this type of psychometric instrument as a “digitally packaged” solution that can be (a) flexibly adapted for specific applications and (b) widely shared within research and clinical communities and rapidly deployed for implementation. This digitally packaged instrument (DPI) framework will expand upon the ideas introduced in this paper by defining a platform upon which a greater diversity of instruments can be constructed in addition to implementing a more general class of software instrument “engines” for presenting instruments and collecting data from participants.

## Conclusions

The MAT software family is a flexible and practical framework for the development and distribution of interactive, video-based assessment tools. The implementation-independent XML document that defines the instrument parameters simplifies the process of defining instruments and variations and reduces the obstacles to modifying and expanding existing instruments as well as simplifying creation of new instruments.

## Availability and requirements

There are two implementations of the MAT software. Both provide nearly identical functionality with regards to the Mobility Assessment Tool. Both implementations are Ⓒ 2011 SeedStage Associates.

### Java Version

**Project home page**: http://mat-sf.wfuhs.arane.us

**Operating system(s)**: Platform independent

**Programming language**: Java

**Other requirements**: Java 1.5 or higher

### iPad Version

**Project home page**: http://ipad-mat.wfuhs.arane.us

**Operating system(s)**: iOS 4.3 or higher

**Programming language**: Objective-C

**Other requirements**: Apple iPad

## Abbreviations

MAT: Mobility assessment tool; IRT: Item response theory; CAT: Computerized adaptive testing; DPI: Digitally packaged instrument

## Competing interests

The authors declare that they have no competing interests.

## Authors’ contributions

RB designed and developed the instrument schema, Java application, and iOS application. AM is a biomechanist and JR is a behavioral psychologist. They are content experts in physical function and mobility disability who contributed to the pilot study design and conduct, development of MAT items, calibration of the instrument, and preparation of this manuscript. AP designed and created the animations. EI is the psychometric expert and co-PI of the study and contributed to the data analysis and validation of the tool. All authors read and approved the final manuscript.

## Pre-publication history

The pre-publication history for this paper can be accessed here:

http://www.biomedcentral.com/1472-6947/13/73/prepub
